# Baseline Red Blood Cell Distribution Width as a Prognostic Marker in High-Risk Resected Cutaneous Melanoma

**DOI:** 10.3390/jcm15051757

**Published:** 2026-02-26

**Authors:** Omer Ekin, Oktay Halit Aktepe

**Affiliations:** 1 Private Practice, Ankara 06800, Türkiye; 2 Department of Medical Oncology, Dokuz Eylul University, Izmir 35330, Türkiye

**Keywords:** biomarker, cutaneous melanoma, prognosis, relapse-free survival, RDW

## Abstract

**Background and Objectives:** High-risk resected cutaneous melanoma carries a substantial risk of recurrence, and additional host-related prognostic biomarkers are needed beyond conventional tumor-centered factors. Red blood cell distribution width (RDW) reflects systemic inflammation and physiological stress and may provide incremental prognostic information. **Materials and Methods:** In this retrospective cohort study, 164 patients with stage II–III cutaneous melanoma who underwent curative-intent surgical resection were analyzed. A receiver operating characteristic (ROC) curve analysis determined the optimal RDW cut-off for relapse-free survival (RFS), which was 14.2%. Patients were categorized into low and high RDW groups accordingly. Survival probabilities were estimated using the Kaplan–Meier method and compared with the log-rank test. Univariate and multivariate Cox proportional hazards regression models were used to evaluate associations between RDW status, clinicopathological variables, and RFS. **Results:** During a median follow-up of 58.3 months, patients with high RDW had significantly shorter RFS compared with those with low RDW. In univariate analysis, elevated RDW was associated with an increased risk of recurrence (HR 2.79, 95% CI 1.39–5.58; *p* = 0.004). After adjustment for key prognostic factors (e.g., stage, Breslow, age, adjuvant therapy), high RDW remained an independent predictor of inferior RFS (HR 2.74, 95% CI 1.37–5.47; *p* = 0.004). Stage III disease also independently predicted worse RFS (HR 4.67, 95% CI 2.04–10.68; *p* < 0.001). **Conclusions:** Baseline RDW independently predicts RFS in high-risk resected stage II–III cutaneous melanoma and may enhance prognostic stratification using a simple, widely available biomarker.

## 1. Introduction

Cutaneous melanoma is an aggressive malignancy, with a pronounced risk of early spread and relapse despite curative surgical treatment [1]. Despite improvements in staging systems and the increasing use of adjuvant systemic therapies, recurrence rates remain clinically significant in stage II–III melanoma [2,3,4]. Prognostic stratification currently relies largely on tumor-related pathological characteristics, such as Breslow thickness, ulceration, and nodal status [2]. However, relapse risk remains heterogeneous among patients with comparable stage and tumor characteristics indicating that tumor-related factors alone do not fully account for clinical outcomes. This highlights an ongoing need for easily accessible biomarkers that reflect host-related biological processes contributing to melanoma progression.

Emerging data suggest that systemic inflammation and immune dysregulation play a central role in melanoma biology [5,6]. As a highly immunogenic malignancy, melanoma progression is determined by a complex interaction between intrinsic tumor biology and host inflammatory processes [7,8]. Systemic inflammatory responses may contribute to tumor progression through multiple mechanisms, including fostering immune escape, enhancing angiogenesis, and facilitating metastatic spread [9,10,11]. Accordingly, peripheral blood–based inflammatory markers have attracted increasing interest as potential prognostic tools in solid tumors including melanoma [12,13,14,15,16,17].

Red blood cell distribution width (RDW), a routinely reported parameter in complete blood count analyses, reflects heterogeneity in erythrocyte size and has traditionally been used in the evaluation of anemia [18]. RDW integrates the cumulative effects of inflammatory cytokines, iron metabolism disturbances, and impaired erythrocyte homeostasis [19,20], making it a biologically plausible marker of cancer-related systemic dysregulation. Across a wide range of solid malignancies, elevated RDW has been associated with inferior survival outcomes [21,22,23]. Taken together, these data indicate that RDW may complement traditional clinicopathological factors in prognostic assessment. Nevertheless, limited evidence exists regarding the prognostic relevance of RDW in melanoma, particularly in high-risk resected stage II–III patients. Thus, we aimed to investigate the prognostic significance of baseline RDW in patients with high-risk resected cutaneous melanoma.

## 2. Materials and Methods

### 2.1. Study Design and Patient Population

This retrospective cohort study included patients with histologically confirmed cutaneous melanoma managed at Dokuz Eylul University (Izmir, Türkiye) and the Omer Ekin Private Clinic (Ankara, Türkiye) between January 2015 and October 2025, including patients referred for follow-up after treatment elsewhere. All patients underwent curative-intent (R0; microscopically margin-negative) surgical resection and had American Joint Committee on Cancer (AJCC) stage II or III disease. Demographic, clinicopathological, laboratory, and follow-up data, including age, sex, primary tumor location, Breslow thickness, Clark level, mitotic rate, ulceration, tumor stage, and receipt of adjuvant therapy, were extracted from electronic medical records. Tumor staging was performed according to the AJCC 8th edition staging system [2]. Eligible patients had available baseline complete blood count data, including RDW, obtained within one month prior to surgery. RDW was recorded as the coefficient of variation (RDW-CV, %), which is the routinely reported RDW parameter in standard complete blood count analyses. Patients were excluded if they had evidence of distant metastatic disease at diagnosis, received neoadjuvant systemic therapy, or had active infection or inflammatory disease at baseline. In addition, immune-mediated systemic autoimmune diseases (including rheumatologic, gastrointestinal/inflammatory bowel disease, neurologic, and autoimmune liver conditions requiring systemic immunosuppression) were excluded, along with hematologic disorders and medications known to substantially affect peripheral blood counts.

### 2.2. Statistical Analysis

Continuous variables are presented as medians with interquartile ranges (IQRs) and were compared using the Mann–Whitney U test. Categorical variables are expressed as counts and percentages and were compared using the chi-square test or Fisher’s exact test, as appropriate. Relapse-free survival (RFS) was defined as the interval from the date of surgical resection to the first documented melanoma recurrence or death from any cause, whichever occurred first. Patients without an event were censored at the date of last follow-up. RFS was estimated using the Kaplan–Meier method and differences between groups were assessed with the log-rank test. Univariate and multivariate Cox proportional hazards regression analyses were performed to evaluate associations between clinicopathological variables, RDW status, and RFS. Results of Cox regression analyses are reported as hazard ratios (HRs) with corresponding 95% confidence intervals (CIs). No formal internal validation procedure (e.g., bootstrapping or cross-validation) was performed for the ROC-derived RDW cut-off; therefore, this threshold should be considered exploratory. In addition, RDW was analyzed as a continuous variable in a separate multivariate Cox proportional hazards model, and the effect size was reported per 1% increase in RDW. Collinearity among covariates included in the RDW models (stage, age, Breslow thickness, adjuvant therapy, and hemoglobin) was assessed using variance inflation factors (VIF), and no problematic collinearity was observed (VIF range: 1.01–1.24). Variables with *p* ≤ 0.20 in univariate analyses were selected for multivariate modeling. All statistical analyses were performed using SPSS software (version 27.0; IBM Corp., Armonk, NY, USA), and a two-sided *p* value < 0.05 was considered statistically significant.

## 3. Results

### 3.1. Baseline Patient Characteristics

A total of 164 patients with high-risk resected cutaneous melanoma were included. Baseline demographic and clinicopathological characteristics of the cohort stratified according to RDW status are summarized in Table 1. The median age of the overall cohort was 61 years (IQR: 49–68), and 93 patients (56.7%) were male. The median Breslow thickness was 3.2 mm (IQR: 1.6–5.9). Ulceration was present in 93 patients (56.7%), and Clark level IV–V disease was observed in approximately 75% of cases. Primary tumor location was most commonly on the extremities (43.3%), followed by the trunk (31.1%), and head and neck region (25.6%). According to pathological staging, 93 patients (56.7%) had stage III disease, while 71 patients (43.3%) were classified as stage II. Adjuvant systemic therapy was administered to 58 patients (35.4%).

**Figure 1:**
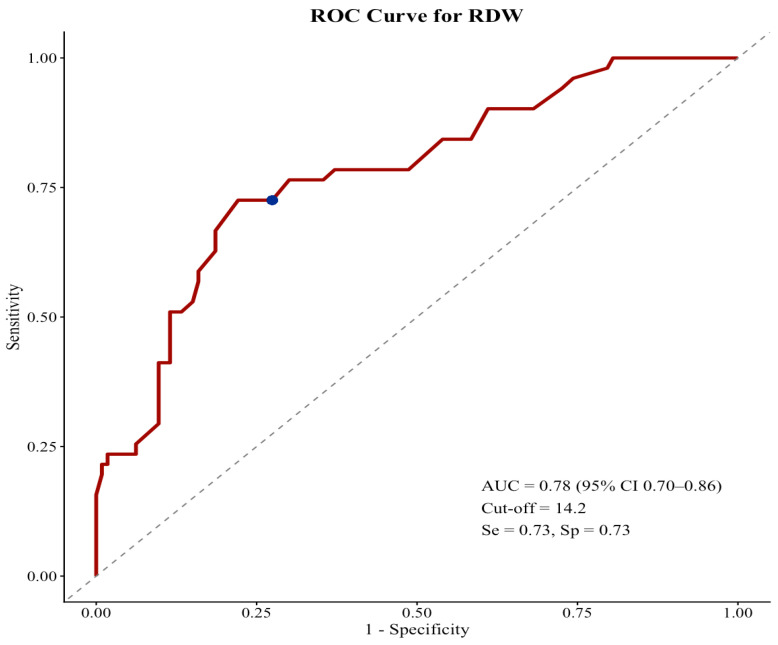
ROC curve of baseline RDW for RFS. The dotted diagonal line represents no-discrimination (AUC = 0.5). The blue dot indicates the optimal RDW cut-off (14.2%), with an AUC of 0.78 (95% CI 0.70–0.86), sensitivity of 0.73, and specificity of 0.73.

ROC analysis for RFS identified an RDW cut-off of 14.2%, yielding an area under the curve (AUC) of 0.78 (95% CI, 0.70–0.86) with both sensitivity and specificity of 0.73 (Figure 1). According to this cut-off value, 96 patients (58.5%) were classified as having low RDW and 68 patients (41.5%) as having high RDW. Baseline hemoglobin (Hb) levels were significantly lower in the high RDW group than in the low-RDW group (median 12.1 [IQR: 10.5–13.5] vs. 13.8 g/dL [IQR: 12.8–14.7], *p* < 0.001). Median age did not differ significantly between patients with low and high RDW (62 vs. 59 years, respectively; *p* = 0.722), and sex distribution was also similar between the two groups (male: 58.3% vs. 54.4%; *p* = 0.618). Tumor-related characteristics were largely comparable between RDW subgroups. The median Breslow thickness was 3.0 mm in the low RDW group and 3.5 mm in the high RDW group (*p* = 0.497). Ulceration was present in 54.2% and 60.3% of patients with low and high RDW, respectively (*p* = 0.435). Clark level IV–V disease was observed in 71.9% of patients with low RDW and 79.4% of those with high RDW (*p* = 0.272). The distribution of primary tumor location did not differ significantly between groups (*p* = 0.546). However, stage III disease was significantly more frequent among patients with high RDW compared with those with low RDW (66.2% vs. 50.0%, respectively, *p* = 0.039). The proportion of patients receiving adjuvant systemic therapy was similar between the low and high RDW groups (33.3% vs. 38.2%, respectively, *p* = 0.518).

### 3.2. RFS and Prognostic Factors

**Figure 2:**
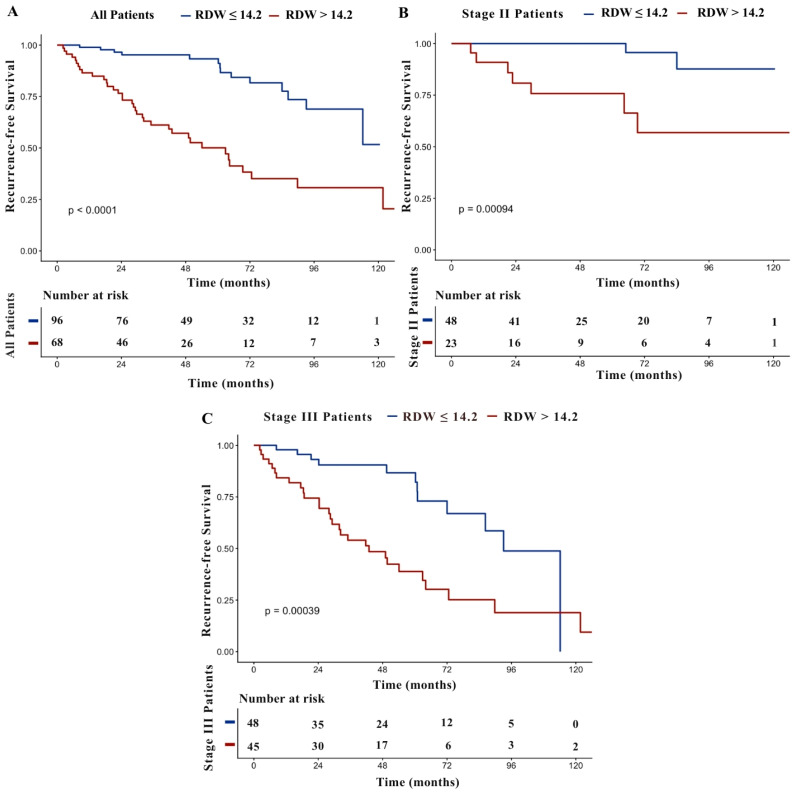
Kaplan–Meier curves for RFS according to RDW status in all patients (**A**), stage II (**B**), and stage III (**C**).

Median follow-up was 58.3 months (95% CI: 47.0–69.6), during which 51 recurrence events were recorded. The median RFS of the overall cohort was 114.2 months (95% CI: 93.5–134.9). In all patients, median RFS differed according to RDW status and was not reached in the low RDW group, compared with 62.9 months (95% CI: 40.4–85.4) in the high RDW group (*p* < 0.001, Figure 2A). When patients were stratified by pathological stage and analyzed according to RDW status, median RFS in the stage II subgroup was not reached in either RDW group, although survival distributions differed significantly (*p* < 0.001, Figure 2B). Within the stage III subgroup, patients with high RDW had a shorter median RFS compared with those with low RDW (42.9 months, 95% CI: 23.8–62.0 vs. 93.1 months, 95% CI: 80.8–105.4, respectively; *p* < 0.001, Figure 2C).

As presented in Table 2, associations between RDW, clinicopathological variables, and RFS were further evaluated using univariate and multivariate Cox regression analyses. In univariate Cox regression analysis, high RDW (vs. low) was associated with inferior RFS (HR: 2.79, 95% CI 1.39–5.58; *p* = 0.004). Additionally, stage III disease (vs. stage II) (HR: 4.90, 95% CI 2.29–10.50; *p* < 0.001) and adjuvant therapy status (No vs. Yes) (HR: 1.78, 95% CI 1.01–3.15; *p* = 0.047) were significantly associated with RFS. In univariate Cox regression, higher Hb levels were associated with improved RFS (per 1 g/dL increase: HR 0.84, 95% CI 0.73–0.97; p = 0.019). However, age (HR: 0.98, 95% CI 0.96–1.00; *p* = 0.108), sex (male vs. female: HR: 1.01, 95% CI 0.57–1.78; *p* = 0.962), Breslow thickness (HR: 1.03, 95% CI 0.98–1.09; *p* = 0.146), and mitotic rate (HR: 1.00, 95% CI 0.99–1.02; *p* = 0.262) were not associated with RFS. In multivariate Cox regression analysis, high RDW (HR: 2.74, 95% CI 1.37–5.47; *p* = 0.004) and stage III disease (HR: 4.67, 95% CI 2.04–10.68; *p* < 0.001) were independent predictors of worse RFS. Age (HR 0.99, 95% CI 0.97–1.01; *p* = 0.608), Breslow thickness (HR: 1.04, 95% CI 0.97–1.11; *p* = 0.252), and adjuvant therapy (No vs. Yes: HR: 1.08, 95% CI 0.59–1.99; *p* = 0.793) were not significant after adjustment. In an additional multivariate Cox regression model in which RDW was analyzed as a continuous variable, RDW remained independently associated with RFS (Table 3). Each 1% increase in RDW was associated with a 19% higher risk of recurrence (HR: 1.19, 95% CI 1.03–1.38; *p* = 0.016). Pathological stage III disease also remained an independent predictor of inferior RFS (HR: 5.19, 95% CI 2.30–11.68; *p* < 0.001).

## 4. Discussion

The present study demonstrates that elevated baseline RDW is an independent prognostic marker for RFS in patients with high-risk resected cutaneous melanoma. When stratified according to a receiver operating characteristic–derived cut-off of 14.2%, elevated RDW was significantly associated with an increased risk of recurrence after adjustment for established clinicopathological prognostic factors, including tumor stage. These observations support existing evidence across solid tumors that RDW may reflect unfavorable host-related biological conditions associated with cancer progression and outcomes [21].

RDW, a measure of anisocytosis, has emerged as a surrogate marker of chronic systemic inflammation, impaired nutritional status, and oxidative stress [24]. Higher RDW values were associated with increased high-sensitivity C-reactive protein and erythrocyte sedimentation rate, both well-recognized inflammatory biomarkers, in a large outpatient population [25]. Additionally, RDW may be associated with circulating cytokines such as interleukin-6, tumor necrosis factor-α, and hepcidin [26,27], which can modulate tumor cell biology. One potential mechanism is that inflammation adversely affects erythropoiesis and red blood cell maturation, which may contribute to increased RDW [23]. These mechanisms are biologically plausible contributors to tumor progression, as chronic inflammation promotes angiogenesis, immune evasion, and metastatic dissemination. In this context, RDW can be considered an integrative biomarker capturing the cumulative effects of inflammation, metabolic dysregulation, and bone marrow stress rather than a simple hematologic parameter.

High RDW has repeatedly been linked to more advanced disease and metastatic burden across solid malignancies. Higher RDW levels in colorectal cancer have been associated with serosal infiltration, nodal metastases, advanced-stage disease, and adhesion to surrounding structures [28]. Similarly, a meta-analysis reported that high RDW was linked to a more aggressive breast cancer phenotype, characterized by larger primary tumors, advanced stage, and higher rates of lymph node metastases [29].

RDW has been widely reported to have prognostic value across multiple solid malignancies. In a meta-analysis including 16 studies and 4267 patients, pooled results demonstrated that elevated RDW was associated with poorer survival outcomes [21]. In lung cancer, RDW has been demonstrated to be a prognostic marker of survival even in patients with early-stage resected disease [30]. RDW has emerged as a prognostic indicator of long-term all-cause mortality in hepatocellular carcinoma, with RDW-based nomograms suggested to refine risk stratification [31]. Similar findings have been reported in endometrial and other gynecologic malignancies, where high RDW predicts both recurrence and overall survival [32,33,34]. In particular, melanoma represents a highly immunogenic cancer in which systemic inflammatory status is a key determinant of disease progression [35]. Although data specifically addressing RDW in melanoma have been limited, emerging evidence supports its prognostic relevance. Hannarici et al. demonstrated that the RDW-to-lymphocyte ratio independently predicted overall survival in cutaneous melanoma [36]. Elevated RDW in our cohort was associated with inferior RFS, highlighting its role as a marker of host–tumor interplay not fully explained by tumor stage alone.

Several limitations of this study should be noted. First, the retrospective, two-center design may introduce selection bias and limit the generalizability of the findings, despite adjustment for key clinicopathological variables. Second, the RDW cut-off value was derived from a single retrospective cohort and was not internally or externally validated. Accordingly, this threshold should be interpreted as exploratory, and its generalizability across different institutions, laboratory platforms, and patient populations may be limited. However, RDW remained prognostic when modeled continuously, supporting a graded rather than threshold-dependent association. Third, RDW was assessed at one preoperative time point, and the prognostic significance of serial RDW measurements during follow-up could not be examined. Fourth, the lack of routine data on systemic inflammatory markers and cytokines limited mechanistic interpretation of the findings. Fifth, stage III disease was more frequent in the high-RDW group, and although pathological stage was included in all multivariate models, some residual confounding related to disease burden may persist, particularly given the modest sample size. Sixth, detailed AJCC stage III substaging was not consistently available, largely due to variability in pathology reporting, particularly among patients referred after initial treatment at outside institutions; this may have limited the granularity of risk adjustment and contributed to residual confounding. Finally, although we adjusted for major clinicopathological prognostic factors available in our dataset, some established melanoma prognostic determinants—such as detailed nodal tumor burden metrics and molecular characteristics including BRAF mutation status—were not uniformly available. These variables were not uniformly available because a proportion of patients were referred after initial management at outside institutions and, particularly in earlier years, detailed nodal pathology reporting and molecular testing were not consistently performed or documented. As a result, residual confounding cannot be excluded, and the observed association between RDW and RFS may be partially influenced if elevated RDW correlates with unmeasured adverse tumor biology or higher nodal burden.

## 5. Conclusions

Baseline RDW is independently associated with RFS in patients with high-risk resected stage II–III cutaneous melanoma. Notably, RDW remained associated with outcomes even after accounting for Hb, indicating that it may reflect broader host-related biological processes beyond anemia. Given its simplicity and wide availability, RDW may represent a readily accessible marker that could complement established prognostic factors. However, these findings are observational and require external validation before RDW can be considered for risk-adapted clinical decision-making.

## Figures and Tables

**Table 1:** Baseline clinical, pathological, and demographic variables stratified according to RDW cut-off.

Characteristics	All Patients (*n* = 164)	Low RDW (*n* = 96, 58.5%)	High RDW (*n* = 68, 41.5%)	*p* Value
Age, years, (IQR)	61 (49–68)	62 (49–69)	59 (49–67)	0.722
Sex				0.618
Female	71 (43.3%)	40 (41.7%)	31 (45.6%)	
Male	93 (56.7%)	56 (58.3%)	37 (54.4%)	
Breslow, (IQR)	3.2 (1.6–5.9)	3 (1.5–5.7)	3.5 (1.9–6.0)	0.497
Clark				0.272
II-III	41 (25%)	27 (28.1%)	14 (20.6%)	
IV-V	123 (75%)	69 (71.9%)	54 (79.4%)	
Mitotic rate, (IQR)	6 (4.0–13.7)	5.5 (3–13.7)	7 (4–13.7)	0.262
Ulceration				0.435
No	71 (43.3%)	44 (45.8%)	27 (39.7%)	
Yes	93 (56.7%)	52 (54.2%)	41 (60.3%)	
Tumor stage				0.039
II	71 (43.3%)	48 (50%)	23 (33.8%)	
III	93 (56.7%)	48 (50%)	45 (66.2%)	
Tumor location				0.546
Trunk	51 (31.1%)	28 (29.2%)	23 (30.3%)	
Extremity	71 (43.3%)	45 (46.9%)	26 (46.2%)	
Head and neck	42 (25.6%)	23 (24%)	19 (23.5%)	
Adjuvant therapy				0.518
No	106 (64.6%)	64 (66.7%)	42 (61.8%)	
Yes	58 (35.4%)	32 (33.3%)	26 (38.2%)	
Hb, g/dL, (IQR)	13.4 (11.7–14.4)	13.8 (12.8–14.7)	12.1 (10.5–13.5)	<0.001

*p* values represent between-group comparisons and were calculated using the chi-square or Fisher’s exact test for categorical variables and the Mann–Whitney U test for continuous variables. Abbreviations: Hb: hemoglobin; IQR: interquartile range; RDW: red blood cell distribution width.

**Table 2:** Univariate and multivariate Cox regression analyses for RFS.

	Univariate		Multivariate	
Variable	HR (95% CI)	*p* Value	HR (95% CI)	*p* Value
Age, years	0.98 (0.96–1.0)	0.108	0.99 (0.97–1.01)	0.608
Gender (male vs. female)	1.01 (0.57–1.78)	0.962	NA	NA
Breslow	1.03 (0.98–1.09)	0.146	1.04 (0.97–1.11)	0.252
Mitotic rate	1.0 (0.99–1.02)	0.262	NA	NA
Stage (III vs. II)	4.90 (2.29–10.50)	<0.001	4.67 (2.04–10.68)	<0.001
Adjuvant therapy (No vs. Yes)	1.78 (1.0–3.15)	0.047	1.08 (0.59–1.99)	0.793
Hb, g/dL	0.84 (0.73–0.97)	0.019	0.92 (0.79–1.06)	0.248
RDW (High vs. low)	2.79 (1.39–5.58)	0.004	2.74 (1.37–5.47)	0.004

Abbreviations: CI: confidence interval; Hb: hemoglobin; HR: hazard ratio; NA: not applicable; RDW: red blood cell distribution width; RFS: relapse-free survival.

**Table 3:** Multivariate Cox regression analysis for RFS with RDW modeled as a continuous variable.

Variable	HR (95% CI)	*p* Value
Age, years	0.99 (0.97–1.02)	0.886
Breslow	1.04 (0.97–1.11)	0.252
Stage (III vs. II)	5.19 (2.30–11.68)	<0.001
Adjuvant therapy (No vs. Yes)	1.24 (0.65–2.36)	0.507
Hb, g/dL	0.89 (0.77–1.03)	0.129
RDW (per 1% increase)	1.19 (1.03–1.38)	0.016

Abbreviations: CI: confidence interval; Hb: hemoglobin; HR: hazard ratio; RDW: red blood cell distribution width; RFS: relapse-free survival.

## Data Availability

The datasets generated and/or analyzed during the current study are available from the corresponding author on reasonable request, subject to patient privacy and ethical considerations.

## Abbreviations

The following abbreviations are used in this manuscript:

AJCC	American Joint Committee on Cancer
AUC	Area Under the Curve
CI	Confidence Interval
Hb	Hemoglobin
HR	Hazard Ratio
IQR	Interquartile Range
NA	Not Applicable
RDW	Red Blood Cell Distribution Width
RFS	Relapse-Free Survival
ROC	Receiver Operating Characteristic
VIF	Variance Inflation Factor
